# M1 Macrophage is a Novel Potential Trigger for Endothelial Senescence: Role of Exosomal miR-155 Targeting SOCS1 Signal

**DOI:** 10.1155/humu/6771390

**Published:** 2025-05-30

**Authors:** Jiang He, Bin Zhang, Hufei Zhang, Qiang Tu, Xi Chen, Yumin Qiu, Zhefu Liu, Wenhao Xia, Xing Wu, Jun Tao

**Affiliations:** ^1^Department of Hypertension and Vascular Disease, National-Guangdong Joint Engineering Laboratory for Diagnosis and Treatment of Vascular Diseases, Key Laboratory of Assisted Circulation, The First Affiliated Hospital, Sun Yat-sen University, Guangzhou, Guangdong, China; ^2^Department of Cardiovascular Disease and Clinical Experimental Center, Jiangmen Central Hospital, Jiangmen, Guangdong, China; ^3^Department of Anesthesiology, The First Affiliated Hospital, Sun Yat-sen University, Guangzhou, Guangdong, China; ^4^Guangxi Hospital Division of the First Affiliated Hospital, Sun Yat-sen University, Nanning, Guangxi, China

**Keywords:** endothelial senescence, exosomes, inflammation, macrophages, miRNA

## Abstract

Age-related proinflammatory microenvironment induced by infiltration of M1 macrophages promotes endothelial senescence-mediated vascular diseases. Macrophages exert their immunomodulatory effects by releasing exosomes. However, the underlying mechanisms governing endothelial cell senescence induced by exosomes derived from M1 macrophages (M1-Exo) remain elusive. In this study, we delved into the intricate interplay between endothelial function and M1 macrophage abundance in the aortas and explored the pivotal role of M1-Exo in endothelial cell senescence and its associated molecular pathways. Our results unveiled a compelling correlation between the infiltration of M1 macrophages in the aortas of aged mice and impaired endothelium-dependent dilatation. Coculturing endothelial cells with M1-Exo engendered the acquisition of a senescent phenotype, marked by increased senescence-associated beta-galactosidase level and a distinct senescence-associated secretory profile. Endothelial cells cocultured with M1-Exo exhibited pronounced signs of cell cycle arrest, accompanied by mitochondrial oxidative damage and dysfunction. Bioinformatics analysis and subsequent validation identified high expression of miR-155 in M1-Exo. The transfer of miR-155 contributed to the prosenescence effect of M1-Exo by targeting SOCS1, subsequently activating JAK2/STAT3 signaling. The administration of M1-Exo into young mice instigated endothelial dysfunction and increased ROS production. Notably, the reduction of miR-155 in M1-Exo partially mitigated such deleterious effects. Our findings demonstrate that exosomal miR-155, originating from M1 macrophages, elicits endothelial cell senescence. The present study brings a groundbreaking insight into the communication between M1 macrophages and endothelial cells as a mediator of vascular aging, providing a promising target for interventions in age-related vascular diseases.

## 1. Introduction

Aging-induced endothelial dysfunction plays a pivotal role in the pathogenesis of age-related ailments, including cardiovascular disease, cerebrovascular disease, and Alzheimer's disease [1–3]. Senescent endothelial cells (ECs) within the arterial wall manifest remarkable gene expression, cell replication, and morphology alterations. These alterations compromise the integrity of the vascular endothelium and contribute to age-related endothelial dysfunction [4–6]. Therefore, extensive investigation is imperative to identify the precise regulatory mechanisms governing endothelial senescence.

Inflammation is a crucial contributor to the aging process [7]. Current data reveal that the inflammatory milieu within the arterial wall plays a vital role in instigating endothelial injury [8, 9]. Macrophages (M*φ*) function as dominant immune cells that actively influence the progression and resolution of this inflammatory milieu. Functionally distinct M*φ* populations emerge through polarization, resulting in two primary subtypes: the cytotoxic M1 phenotype, which was traditionally activated, proinflammatory, and the M2 phenotype, which was alternatively activated, anti-inflammatory [10]. Recent research has additionally demonstrated that M*φ* exist on a continuum with diverse phenotypes beyond this simplified M1/M2 dichotomy, displaying remarkable plasticity and mixed phenotypes in response to complex tissue microenvironments [11]. Previous studies have documented a significant infiltration of M1-polarized M*φ* into the arterial wall during the aging process [12, 13]. However, whether M1 M*φ* exacerbate endothelial senescence remains to be determined, requiring further clarification of the underlying mechanisms.

Exosomes (Exo) are small extracellular vesicles (30–150 nm in diameter) secreted from different cell types, such as M*φ*, containing a variety of biomolecular cargos including proteins, nucleic acids, and liquids [14, 15]. MicroRNAs (miRNAs), short noncoding RNA transcripts approximately 22 nucleotides in length, have been recognized as key mediators of Exo-induced biological effects [16, 17]. Recent research demonstrated that transmission of miR-155 by Exo generated from M1 M*φ* suppressed angiogenesis and exacerbated cardiac dysfunction [18]. Moreover, M1 M*φ*-derived Exo transport miR-146a and miR-146b to inhibit the migratory and invasive capabilities of trophoblast cells [19]. Recent studies have highlighted the critical roles of exosomal miRNAs in aging processes, where they serve as crucial mediators of intercellular communication and potential biomarkers for age-related pathologies [20]. Exosomal miRNAs can regulate multiple aspects of vascular aging, including endothelial dysfunction, senescence, and inflammation. However, the specific role of M1 M*φ*-derived Exo in accelerating endothelial senescence remains elusive.

Accumulating evidence indicates that senescence is marked by cell cycle arrest and mitochondrial dysfunction [21–23]. Senescent cells demonstrate increased expression of genes that enforce the cell cycle arrest in G1 phase, notably p21Cip1, which has been linked to inducing endothelial senescence [24, 25]. Mitochondrial dysfunction, characterized by reduced respiratory capacity and elevated reactive oxygen species (ROS) production, is a cause and consequence of cellular senescence [22]. Dysfunctional mitochondria and excessive mitochondrial reactive oxygen species (mtROS) production contribute to endothelial senescence and vascular aging [26, 27].

In this study, we investigated whether Exo derived from infiltrated M1 M*φ* in arterial wall aggravate endothelial senescence by modulating cell cycle arrest and mitochondrial oxidative damage. Additionally, we sought to explore the functional miRNA harbored within Exo derived from M1 M*φ* and uncover the relevant molecular pathways. The current investigation brings a groundbreaking perspective on the interplay between M1 M*φ* and ECs, elucidating their role as a pivotal mediator of vascular aging. This newfound understanding provides a potential novel target for interventions in age-related vascular diseases.

## 2. Materials and Methods

### 2.1. Animals

All animal experimental procedures were performed with approval from the Ethics Committee of the First Affiliated Hospital of Sun Yat-sen University and in accordance with the Guide for the Care and Use of Laboratory Animals published by the National Institutes of Health (NIH Publication Eighth Edition, updated 2011). Beijing Vital River Experimental Animal Technology Co. Ltd. (China) supplied the C57BL/6J mice at different ages (3 and 20 months) used in this study.

### 2.2. Vascular Functional Study

After animals were sacrificed, the thoracic aortas were rapidly excised and placed in an oxygenated ice-cold Krebs–Henseleit solution. For the measurement of isometric tension changes, aortic segments from the mice were attached to a wire myograph system (Danish Myo Technology, Aarhus, Denmark). Aortic segments precontracted with phenylephrine (Phe, 3 *μ*mol/L) to establish stable baseline tension were subsequently exposed to increasing concentrations of acetylcholine (ACh, 3 nmol/L–10 *μ*mol/L) to assess endothelium-mediated relaxation.

For flow-mediated dilatation (FMD) assessment, second-order resistance mesenteric artery segments were carefully isolated from the surrounding adipose tissue in sterile phosphate-buffered saline (PBS) and then cultivated in Dulbecco's modified Eagle medium (DMEM) for 48 h. Individual artery segment was mounted on a pressure myograph by cannulating between two glass cannulas. Changes in the vessel diameter and intraluminal pressure were continuously recorded via a video camera–equipped light-inverted microscope (Model 110P, Zeiss Axiovert 40) and analyzed with imaging software (Myo-View, Danish Myo Technology). Arterial segments were maintained under an intraluminal pressure of 80 mmHg with continuous Phe administration (10 *μ*mol/L) to establish sustained vasoconstriction. FMD was triggered by a pressure shift equal to generating shear stress levels approximately 15 dynes/cm^2^. At the experimental endpoint, maximum passive dilatation was reached at the end of the experiment by substituting the perfusion solution with Ca^2+^-depleted Krebs buffer with 2 mM EGTA. FMD was presented as percentage of diameter changes: (FMD − Phe tone)/(passive dilatation − Phe tone).

### 2.3. Cell Culture

The primary bone marrow–derived macrophages (BMDMs) were obtained and cultured according to previously established methods [28]. The femur and tibia of 6-week-old mice were excised and placed in a 100-mm cell culture dish containing RPMI 1640 complete media. Bone marrow was then flushed into a new cell culture dish containing RPMI 1640 complete media. Following centrifugation at 400 × *g* for 5 min, the cell pellet was reconstituted in L929-conditioned M*φ* media. M1 polarization was induced by treating BMDMs with IFN-*γ* (215 U/mL) and LPS (10 ng/mL, added 8 h after the start of IFN-*γ* treatment) for 24 h. Measurement of M1 M*φ* markers by real-time polymerase chain reaction (RT-PCR) and immunofluorescence was used to identify polarization.

The mouse aortic EC line was acquired from the Type Culture Collection of the Chinese Academy of Sciences (Shanghai, China). ECs were cultured in DMEM (Gibco, Thermo Fisher, United States) supplemented with 10% FBS and 1% penicillin–streptomycin at 37°C and 5% CO_2_.

### 2.4. Exo Extraction and Identification

Exo were extracted from the supernatant of BMDMs that were pretreated with or without LPS/IFN-*γ*. Before collecting the culture medium, the BMDMs underwent two washes with PBS. After 48 h of LPS/IFN-*γ* stimulation, the medium was changed to a medium without Exo. Exo were isolated using differential centrifugation following the protocol established by Yin et al. [29]. The culture medium was subjected to sequential centrifugation steps of 500 × *g* (2 × 10 min), 2000 × *g* (1 × 20 min), and 10,000 × *g* (1 × 30 min) to remove BMDMs, cell debris, and vesicles with larger sizes. The supernatants were subsequently filtered through 0.22-*μ*m filter units (Millex-GP, Millipore, Germany) and subjected to ultracentrifugation at 100,000 × *g* for 3 h at 4°C. Following the removal of the supernatant, the ice-cold PBS was used to resuspend the precipitated pellets. The suspension underwent a second ultracentrifugation (100,000 × *g*, 3 h, 4°C). Exo precipitates were reconstituted in PBS and stored at −80°C.

### 2.5. Transmission Electron Microscopy (TEM)

TEM was used to examine the morphology of Exo. The Exo sample was first suspended in 2.5% glutaraldehyde for 2 h and rinsed with PBS. Then, 20 *μ*L of Exo suspension was applied to a copper grid (200-mesh, coated by formvar carbon film, Thermo Fisher). The Exo were then stained with 2% (*v*/*v*) uranyl acetate and air-dried for 10 min. The stained grids were subsequently visualized using a Tecnai G2 Spirit TEM (Zeiss, Oberkochen, Germany) at 120 kV.

### 2.6. Exo Internalization Assay

In accordance with the manufacturer's guidelines, red fluorescent PKH67 dye (Sigma) was used to label M1-Exo. Subsequently, the labeled Exo were added to ECs and maintained in coculture conditions for various time intervals (30 min, 2, 4, and 6 h). ECs were rinsed with PBS followed by fixation using paraformaldehyde (4%) for 15 min. Nuclei were stained with DAPI, and imaging was conducted using a confocal laser scanning microscope (Carl Zeiss, LSM780).

### 2.7. Senescence-Associated-*β*-Galactosidase (SA*β*G)

SA*β*G staining was performed utilizing a SA-*β*-gal Detection Kit (Beyotime). Cells were harvested and rinsed with PBS, followed by a 15-min incubation in a fixative solution. Subsequently, they were stained with a SA*β*G reaction solution at 37°C for 16 h. The blue-stained cells were recognized as senescent cells, and the total count of senescent cells was quantified under a microscope across 15 fields of view.

### 2.8. Immunofluorescence Staining

For aortic tissues, frozen sections of the aortas were thawed at room temperature and rinsed with PBS. After incubation with a blocking solution, frozen sections of the aortas were incubated overnight with anti-CD68 at 4°C. The following day, these specimens were incubated with a secondary antibody and subsequently counterstained with DAPI. The secondary antibody used in this experiment was purchased from Sino Biological (China).

To prepare cell samples, cultured cells were subjected to fixation in methanol (ice-cold, 10 min, 4°C), followed by three consecutive rinses with PBS. The fixed cell monolayers were permeabilizated through incubation with a permeabilization agent (0.1% Triton X-100 dissolved in PBS, 10 min, room temperature). To reduce nonspecific signal, samples were treated for 30 min with a blocking solution composed of 0.5% Tween-20 and 1% BSA in PBS. Subsequent incubation with the primary antibodies, detailed in Table S2, was carried out at 4°C overnight. The secondary antibody incubation was conducted for 60 min at room temperature. DAPI was used to label the nuclei.

Fluorescence images were obtained using an LSM780 confocal laser scanning microscope (Carl Zeiss) equipped with a Plan-Apochromat 63x/1.4NA oil DIC M27 objective. Image processing was conducted utilizing Zen-Lite 2010 software (Carl Zeiss).

### 2.9. miR-155-5p Mimic or Inhibitor Transfection

The miR-155-5p mimic, inhibitor, and corresponding negative control (miR-Control) were obtained from MedChemExpress (Monmouth Junction, New Jersey, United States). 1 × 10^6^ M1 M*φ* were seeded onto a 10-cm culture dish and maintained for 24 h. Following this, transfection was performed by introducing 50-nM concentrations of miR-155-5p mimic, inhibitor, or miR-Control using Lipofectamine 2000 (Invitrogen). The transfection process was carried out under standard cell culture conditions of 48 h at 37°C in a 5% CO_2_ incubator. Following transfection, culture supernatants were collected, and Exo were isolated in accordance with previously established methods.

### 2.10. mtROS

The generation of mtROS in ECs was assessed using the MitoSOX probe. The ECs from each experimental condition were seeded onto six-well plates at a density of 3 × 10^5^ cells per well in the culture medium. Upon achieving 80% confluence, ECs were incubated with Exo. Then, cells were treated with a MitoSOX probe (37°C, 30 min). Subsequently, the labeled cells underwent three washes with PBS. Quantitative assessment of mtROS generation was performed via flow cytometric measurement (CytoFLEX, Beckman Coulter, United States), and qualitative visualization of the fluorescent MitoSOX signal was achieved through confocal laser microscopy (LSM780, Carl Zeiss).

### 2.11. Flow Cytometry Analysis of M*φ* Infiltrated in the Aortas

To prepare a single-cell suspension of aortic cells, the entire aorta was excised during the development of aortic dissection, and all surrounding connective and adipose tissues were completely removed. The aortas were sectioned and enzymatically digested at 37°C for 90 min using 1 mL of dissociation enzyme solution (Sigma-Aldrich, St. Louis, United States) to obtain single cells.

Flow cytometry was utilized to identify proinflammatory M*φ* using APC anti-CD11c, FITC anti-CD45, BV421 anti-F4/80, and PE anti-CD206 antibodies (Table S2). The gating strategy involved isolating CD45+F4/80+ cells from single-cell suspensions of the entire aorta. The proportion of CD11c+CD206- cells within CD45+F4/80+ cells was subsequently assessed to determine M1 M*φ* activation. Cells were incubated with primary antibodies for 30 min on ice. After washing out the unbound antibodies, labeled cells were analyzed using a CytoFLEX (Beckman Coulter, United States). Over 600,000 cells were collected for analysis, with number of CD45+ cells exceeding 5000. Data analysis was conducted using FlowJo V10 software (Tree Star Inc., Ashland, Oregon, United States).

### 2.12. RT-PCR

Total RNA was isolated from cultured cells and mouse tissues using the TRIzol reagent (TransGen Biotech, China). The concentration and purity of the RNA samples were evaluated by spectrophotometric analysis at wavelengths of 260 and 280 nm. cDNA synthesis was performed utilizing a reverse transcriptase kit (TransGen Biotech, China). The relative expression levels of mRNA were measured and analyzed using qRT-PCR SuperMix (TransGen Biotech, China). GAPDH was utilized as the reference gene for normalizing mRNA, while snRU6 served as the reference for miRNA analysis. Details of the primer sequences are provided in Table S1.

### 2.13. Western Blotting

Frozen tissue, cultured cells, or Exo were lysed using RIPA butter (Beyotime Biotechnology, Shanghai, China). Protein sample (5 *μ*g) was initially separated by SDS-PAGE (10% separation gel) and subsequently transferred to a PVDF membrane. Membranes were then immersed in a blocking buffer (5% nonfat milk, 1 h, room temperature) to prevent nonspecific interactions. Primary antibody incubation was conducted overnight at 4°C with gentle agitation. Following a 1-h incubation with secondary antibodies at room temperature, the chemiluminescent signals were detected by ECL reagent and analyzed with ImageJ software (NIH). *β*-Actin (ACTB) was used as a loading control to normalize protein expression. Detailed primary antibodies' information is provided in Table S2. Secondary antibodies were obtained from Sino Biological (China).

### 2.14. Mitochondrial Respiration Analysis

Oxygen consumption rate (OCR) measurements were evaluated using the Seahorse XF Cell Mito Stress Test Kit. Cartridges were hydrated overnight in HPLC-grade water within a CO_2_-free incubator for each measurement. ECs were plated in XF96 microplates (Seahorse Bioscience) at a density of 5000 cells/well and incubated overnight to ensure adherence. Culture medium was subsequently substituted with XF base medium (pH 7.4 DMEM, 1-mM pyruvate, 2-mM glutamine, and 10-mM glucose) and equilibrated for 1 h in a 37°C CO_2_-free incubator. Finally, microplate was loaded into the Agilent Seahorse Bioscience XF96 Extracellular Flux Analyzer (Agilent Technologies AG, Basel, Switzerland). The OCR rates of mitochondrial oxidative phosphorylation (OXPHOS) and glycolysis flux were quantified by sequentially injecting 3-*μ*M oligomycin (ATP synthase inhibitor), 3-*μ*M FCCP (mitochondrial uncoupler), and 2-*μ*M rotenone/antimycin A (complex I/III inhibitors).

### 2.15. The Administration of Exo In Vivo

Three-month-old C57BL/6J mice were randomly allocated to the young, young + Exo, and young + miR-155 inhibitor-Exo (Exo derived from miR-155 inhibitor-treated M1 M*φ*) groups, and a group of 20-month-old aged mice was established. Six mice were included in each group. In the young + Exo and young + miR-155 inhibitor-Exo groups, 20 *μ*g Exo was intravenously injected into mice [30]. Exo were administrated every 5 days for a total of 40 days. The evaluation of endothelial function was conducted 40 days after Exo were administered via intravenous injection into the tail vein.

### 2.16. Statistical Analysis

All statistical data were presented as mean ± standard deviation (s.d.) of *n* independent experiments. Intergroup comparisons were analyzed using a two-tailed unpaired Student's *t*-test. For analyses involving multiple groups, statistical significance was determined using one-way ANOVA followed by Bonferroni's post hoc test. *p* values less than 0.05 (*p* < 0.05) were considered statistically significant. Statistical analysis and scatter plots were carried out with GraphPad Prism 9.0 and SPSS 29.0 software (SPSS, Chicago, Illinois, United States).

## 3. Results

### 3.1. Impaired Endothelial Function Is Associated With Infiltration of M1-Polarized M*φ*

**Figure 1:**
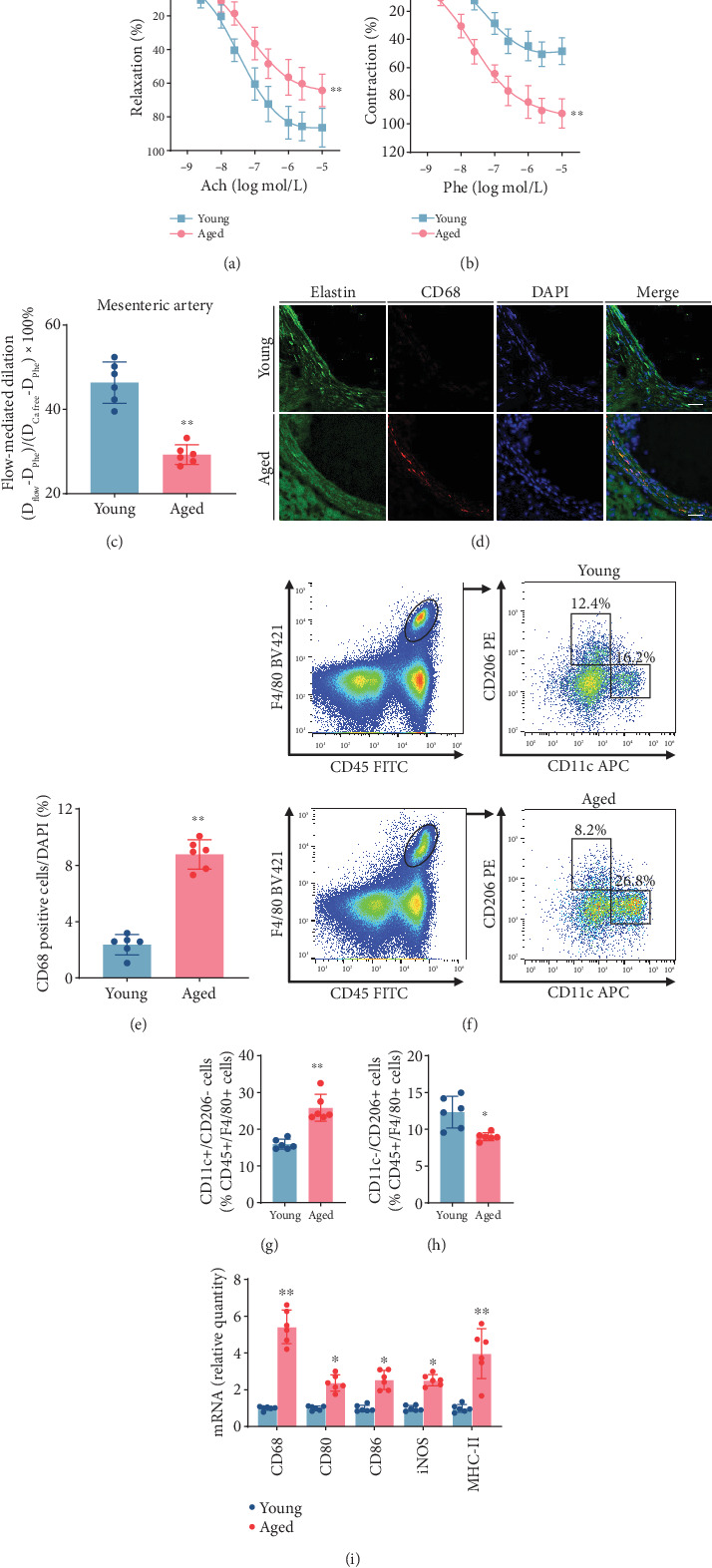
M1-polarized macrophages infiltrated in aortas from old mice were associated with endothelial dysfunction. (a) ACh concentration–response curves showed impaired endothelial-dependent relaxation in old mouse aortas (*n* = 6, biological replicates). (b) Cumulative contraction–response curves to phenylephrine exhibited more significant contractions in old mouse aortas (*n* = 6, biological replicates). (c) Flow-mediated dilatation was reduced in aged mouse mesenteric arteries. (d) Immunofluorescence staining for macrophages (CD68, red) and elastin (green) in transverse cryosections of aortas from young and aged mice with aortic dissection (scale bar, 40 *μ*m). (e) Quantification of CD68-positive cells/DAPI (*n* = 6, biological replicates). (f–h) Flow cytometry analysis and quantification of the percentage of CD11c+/CD206− or CD11c-/CD206+ macrophages among F4/80+CD45+ cells in the aorta from young and aged mice (*n* = 6, biological replicates). (i) The mRNA levels of the M1 macrophage markers in the aorta from young and aged mice were analyzed by qRT-PCR (*n* = 6, biological replicates). ⁣^∗^*p* < 0.05 and ⁣^∗∗^*p* < 0.01 versus young.

To explore the association between M1 M*φ* infiltrated in arterial wall and endothelial dysfunction during aging process, we utilized a murine model of advanced age at 20 months. Our results revealed a notable reduction in ACh-induced endothelium-dependent relaxations in the aortas of aged mice (20-month-old) (Figure 1a). Additionally, the aortic rings of aged mice exhibited more pronounced contractions to cumulative doses of Phe compared to the young mice (3-month-old) (Figure 1b). Similarly, diminished FMD was observed in small resistance arteries of aged mice (Figure 1c).

Subsequently, we assessed the abundance of infiltrated M1 M*φ* by conducting immunofluorescent staining of aortic sections from young and aged mice using specific markers, CD68. Immunofluorescence experiments unveiled a conspicuous augmentation in the number of CD68+ cells that infiltrated the arterial walls of aged mice in contrast to young mice (Figure 1d,e). Flow cytometric analysis, employing gating strategies based on CD45, F4/80, CD11c, and CD206 expression, was conducted to assess M*φ* polarization (Figure 1f). The percentage of CD11c+/CD206− M*φ*, characterized as M1 M*φ*, was elevated within the F4/80+CD45+ cell population. In contrast, the level of M2-polarized M*φ*, defined as CD11c-/CD206+ within the F4/80+CD45+ cell population, was decreased in the aortas of aged mice (Figure 1g,h). Furthermore, we observed statistically significant differences in the mRNA expression levels of M1 M*φ* markers, including CD68, CD80, CD86, iNOS, and MHC-II, between young and aged mice (Figure 1i). Overall, these results suggest that an increase in M1 M*φ* infiltration accompanied the impaired endothelial function of old mice.

### 3.2. Characterization of Exo Derived From M1 M*φ*

Initially, we separated and differentiated BMDMs into M1 M*φ* by stimulating them with LPS and IFN-*γ* (Figure S1A). Remarkably, the morphological transformation of BMDMs from a rounded form to an elongated and spindle-shaped appearance was observed following exposure to LPS and IFN-*γ* (Figure S1B). Furthermore, immunofluorescence staining demonstrated a noticeable increase iNOS and CD11c intensity following LPS and IFN-*γ* treatment (Figure S1C). Likewise, there was a marked upregulation in the mRNA expression levels of M1 M*φ* markers, including Tnfa, iNOS, Il1b, and Il6, in the group subjected to LPS and IFN-*γ* stimulation (Figure S1D). These findings indicated the successful differentiation of BMDMs into M1 M*φ* upon stimulation with LPS and IFN-*γ*.

Subsequently, we focus on isolating and identifying Exo secreted by M1 M*φ* from the culture medium. NTA (Figure S2A, B) and TEM (Figure S2C) revealed that M1-Exo displayed a distinct cup-shaped morphology with an average diameter of 114 nm. Western blot analysis revealed that the characteristic markers CD63, CD81, and TSG101 were highly expressed in M1-Exo (Figure S2D). Collectively, we successfully isolated and purified Exo secreted by M1 M*φ*, paving the way for further investigations.

### 3.3. M1 M*φ*-Derived Exo Aggravate EC Senescence

**Figure 2:**
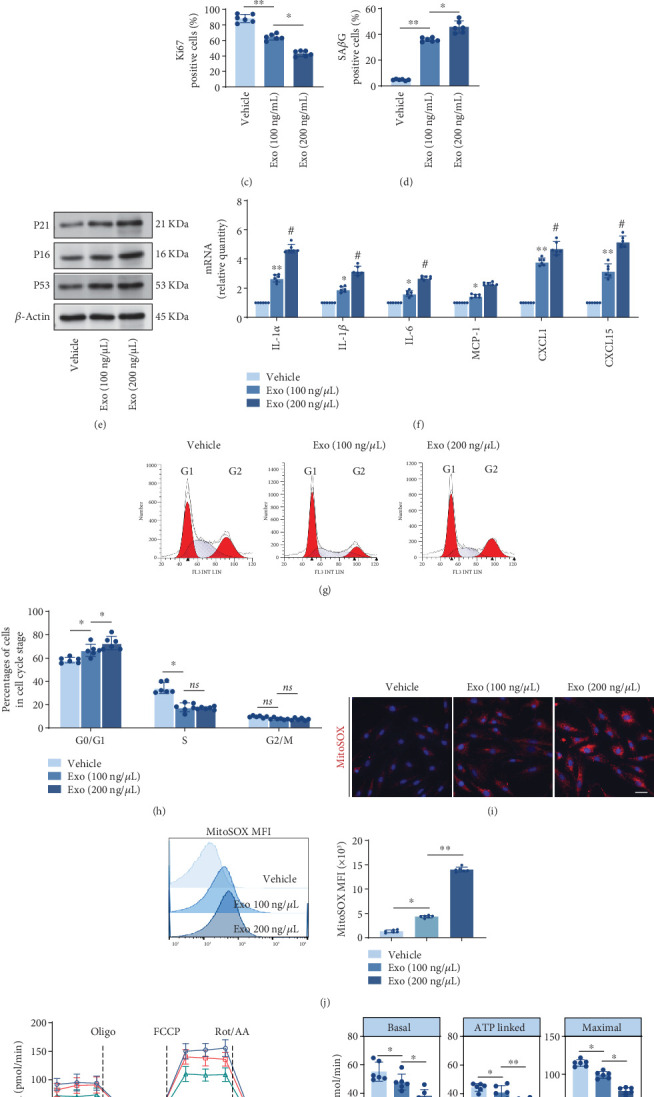
M1 macrophage-derived exosomes aggravated endothelial senescence via inducing cell cycle arrest and mitochondrial oxidative damage. (a) Immunofluorescence staining of Ki67 and DAPI in ECs with different concentrations of M1-Exo (100, 200 ng/*μ*L) coincubation (*n* = 6). Scale bar, 20 *μ*m. (b) Representative images of SA*β*G staining in ECs with or without M1-Exo coincubation (*n* = 6, biological replicates). Scale bar, 100 *μ*m. (c) Quantitation of Ki67-positive cells of (a). (d) Quantitation of SA*β*G staining in ECs of (b). (e) Representative western blot images and quantification of the senescence markers P16, P21, and P53 in ECs with different concentrations of M1-Exo (100, 200 ng/*μ*L) coincubation (*n* = 6, biological replicates). (f) The relative mRNA abundance of key SASP components in ECs with different concentrations of M1-Exo (100, 200 ng/*μ*L) coincubation (*n* = 6, biological replicates). ⁣^∗^*p* < 0.05 and ⁣^∗∗^*p* < 0.01 versus vehicle; ^#^*p* < 0.05 and ^##^*p* < 0.01 versus Exo (100 ng/*μ*L). (g, h) Cell cycle analysis of ECs transfected with different concentrations of M1 M*φ*-derived exosomes (M1-Exo) (100, 200 ng/*μ*L) coincubation by flow cytometer (*n* = 6, biological replicates). (i) Fluorescence staining of mitochondrial ROS production indicated by MitoSOX probe in ECs transfected with different concentrations of M1-Exo (100, 200 ng/*μ*L) (*n* = 6, biological replicates). Scale bar, 20 *μ*m. (j) Flow cytometry analysis of mitochondrial ROS production indicated by MitoSOX probe in ECs transfected with different concentrations of M1-Exo (100, 200 ng/*μ*L) coincubation (*n* = 6, biological replicates). (k) Mitochondrial respiratory capacity of ECs transfected with different concentrations of M1-Exo (100, 200 ng/*μ*L) coincubation measured by O_2_ consumption rate (OCR) using a Seahorse analyzer and quantification of basal, ATP-linked, and maximal OCR (*n* = 6, biological replicates). ⁣^∗^*p* < 0.05 and ⁣^∗∗^*p* < 0.01.

We further investigated the influence of various concentrations of M1-Exo on EC senescence in vitro. After a 6-h exposure to PKH67-labeled Exo, red fluorescence emerged within the ECs, indicating the internalization of M1-Exo by ECs (Figure S2E). Since cellular senescence is primarily characterized by the arrest of cell proliferation, we then assessed the expression of Ki67, a marker for proliferating cells. The percentages of Ki67-positive cells markedly decreased in the presence of M1-Exo coincubated (Figure 2a,c). The data presented in Figure S3 and Figure 2b,d collectively demonstrated that M1-Exo induced EC senescence, as evidenced by the increased SA*β*G level, a classical method for detecting cellular senescence. Moreover, stimulation with M1-Exo resulted in a significant upregulation of senescence-associated markers, including the cell cycle regulator p16INK4a, p21Cip1, and p53 (Figure 2e and Figure S4). As cellular senescence is linked to a unique secretory profile, referred to as the senescence-associated secretory phenotype (SASP), we examined the classical key SASP components in ECs with or without M1-Exo treatment. Remarkably, M1-Exo treatment led to increased mRNA levels of IL-1*α*, IL-1*β*, IL-6, MCP-1, CXCL1, and CXCL15 (Figure 2f). Taken together, our findings suggest that M1-Exo markedly exacerbate EC senescence.

Cellular senescence is characterized by an irreversible arrest of the cell cycle in the G1 phase [23, 24] and an increased production of ROS, primarily attributed to mitochondria dysfunction [22]. Hence, we sought to investigate whether M1-Exo induced cell cycle arrest and mitochondrial oxidative damage in ECs. Flow cytometry analyses revealed a G0/G1 phase arrest in the cell cycle when ECs were exposed to M1-Exo (Figure 2g,h). MitoSOX Red was employed to detect the mtROS levels of ECs. Both confocal imaging and flow cytometry convincingly demonstrated that M1-Exo triggered excessive production of mtROS (Figure 2i,j). To assess the impact of M1-Exo on mitochondrial function, particularly OXPHOS, we conducted the Seahorse Mito Stress Test to obtain baseline, oligomycin-inhibited, FCCP-activated, and rotenone/antimycin-inhibited OCR values in ECs with or without M1-Exo treated. As shown in Figure 2k, the M1-Exo-treated ECs exhibited significantly lower basal, ATP-linked, and maximal OCR values, indicating impaired mitochondrial OXPHOS in response to M1-Exo. These findings provided compelling evidence that M1-Exo instigates cellular senescence in ECs by inducing cell cycle arrest and mitochondrial oxidative damage.

### 3.4. miR-155 Mediates the Prosenescence Effect of M1 M*φ*-Derived Exo

**Figure 3:**
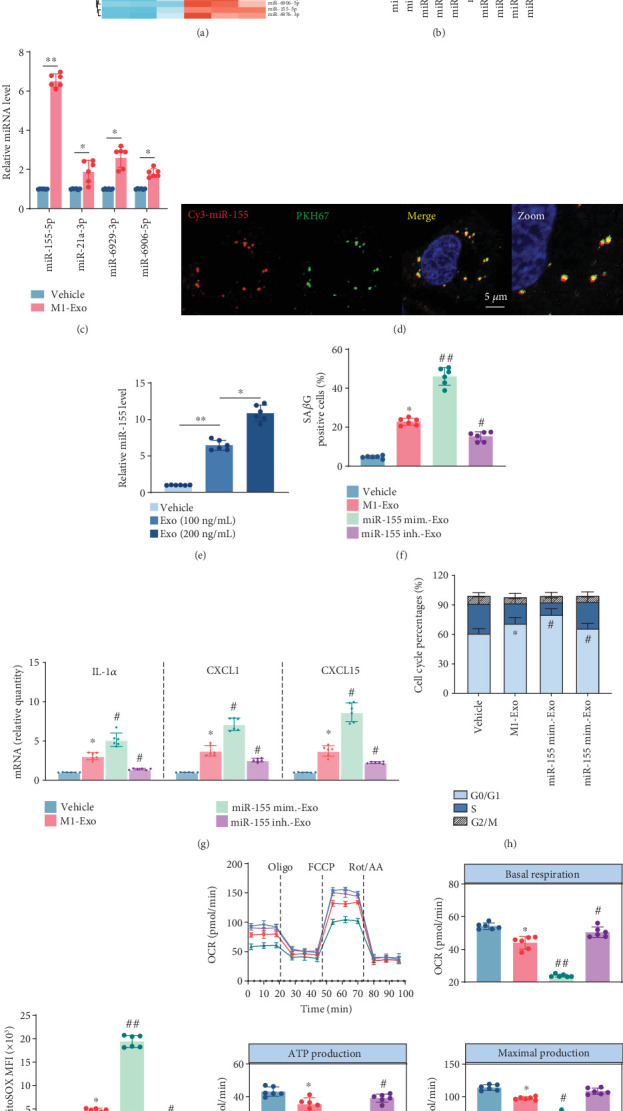
M1 macrophage-derived exosomes result in endothelial senescence by delivery of miR-155. (a) Heatmap of the differential expression of microRNAs in GSE143845. (b) The expression levels of upregulated miRNAs in (a) were measured in M0 and M1 M*φ*-derived exosomes by qRT-PCR. (c) The expression levels of the top four miRNAs in (b) were measured in ECs with or without M1-Exo coincubation (*n* = 6, biological replicates). ⁣^∗^*p* < 0.05 and ⁣^∗∗^*p* < 0.01. (d) Confocal microscope images showed the delivery of miR-155 to ECs by PKH67-labeled exosomes. miR-155 was labeled by Cy3. Scale bar, 5 *μ*m. (e) The expression levels of miR-155 in ECs transfected with different concentrations of M1-Exo (100, 200 ng/*μ*L) coincubation (*n* = 6, biological replicates). ⁣^∗^*p* < 0.05 and ⁣^∗∗^*p* < 0.01. M1-Exo, miR-155 mim.-Exo, and miR-155 inh.-Exo (exosomes derived from miR-155 mimic or miR-155 inhibitor-treated M1 macrophage) were prepared and cocultured with ECs. (f) The percentage of SA-*β*-gal-positive cells (g) and the relative mRNA abundance of key SASP components, IL-1*α*, CXCL1, and CXCL15, were measured in ECs. (h) Cell cycle analysis of ECs cocultured with M1-Exo, miR-155 mim.-Exo, miR-155 inh.-Exo, and PBS, respectively. (i) Quantification of MitoSOX probe-indicated mtROS production by flow cytometry in ECs cocultured with M1-Exo, miR-155 mim.-Exo, miR-155 inh.-Exo, and PBS, respectively. (j) OCR was used to measure the mitochondrial respiratory capacity of ECs cocultured with M1-Exo, miR-155 mim.-Exo, miR-155 inh.-Exo, and PBS, respectively, and quantification of basal, ATP-linked, and maximal OCR (*n* = 6, biological replicates). ⁣^∗^*p* < 0.05 and ⁣^∗∗^*p* < 0.01 versus vehicle. ^#^*p* < 0.05 and ^##^*p* < 0.01 versus M1-Exo.

Accumulating evidence has firmly established the pivotal role of miRNAs encapsulated within Exo in regulating cellular function. In light of this, our current investigation is aimed at elucidating the potential involvement of miRNAs in mediating the exacerbation of endothelial senescence by M1-Exo. Based on bioinformatics analysis of GSE143845, a miRNA profile in RAW264.7 cells treated without or with LPS and IFN*γ*, we identified the most abundant miRNAs within M1 M*φ* (Figure 3a) and compared the expression of the top 10 miRNAs in M0 or M1-Exo using qRT-PCR (Figure 3b). Among these, we selected the miRNAs that ranked within the top four in Figure 3b and assessed their expression in ECs with or without exposure to M1-Exo. Notably, we observed a significant increase in miR-155 expression within M1-Exo-treated ECs (Figure 3c). By incubation with PKH67-labeled Exo derived from Cy3-labeled miR-155 M1 M*φ*, we discovered a colocalization of the Cy3-miR-155 and PKH67 signals within the cytoplasm of ECs (Figure 3d), strongly suggesting that miR-155 could indeed be transferred from M1 M*φ* to ECs via Exo. Furthermore, as shown in Figure 3e, M1-Exo induced upregulation of miR-155 in a concentration-dependent manner.

To further explore the role of miR-155 in the regulatory influence of M1-Exo on endothelial senescence, we constructed miR-155 overexpression and knockdown M1 M*φ* through transfection with a miR-155 mimic and inhibitor, subsequently obtaining their respective Exo. Transfection efficiency is shown in Figure S5. Exo derived from miR-155 mimic-treated M1 M*φ* (miR-155 mimic-Exo) significantly increased miR-155 expression within the target ECs, while Exo derived from miR-155 inhibitor-treated M1 M*φ* (miR-155 inhibitor-Exo) exerted an opposite effect. The miR-155 mimic-Exo group displayed a striking augmentation in SA*β*G activity (Figure 3f), alongside a substantial elevation in the mRNA levels of crucial SASP factors (Figure 3g). Conversely, knockdown of miR-155 mitigated such effects. miR-155 mimic-Exo significantly arrested ECs in the G1 phase of cell cycle (Figure 3h). Overexpression of miR-155 was accompanied by a pronounced escalation in mtROS generation (Figure 3i), ultimately resulting in substantial impairment of mitochondrial OXPHOS within ECs (Figure 3j). Conversely, treatment with miR-155 inhibitor-Exo yielded opposite outcomes. Collectively, these findings elucidate the pivotal role of miR-155 within M1-Exo in instigating EC senescence, primarily through the induction of cell cycle arrest and mitochondrial oxidative damage.

### 3.5. miR-155 Negatively Targets SOCS1 and Activates the JAK2/STAT3 Signaling

**Figure 4:**
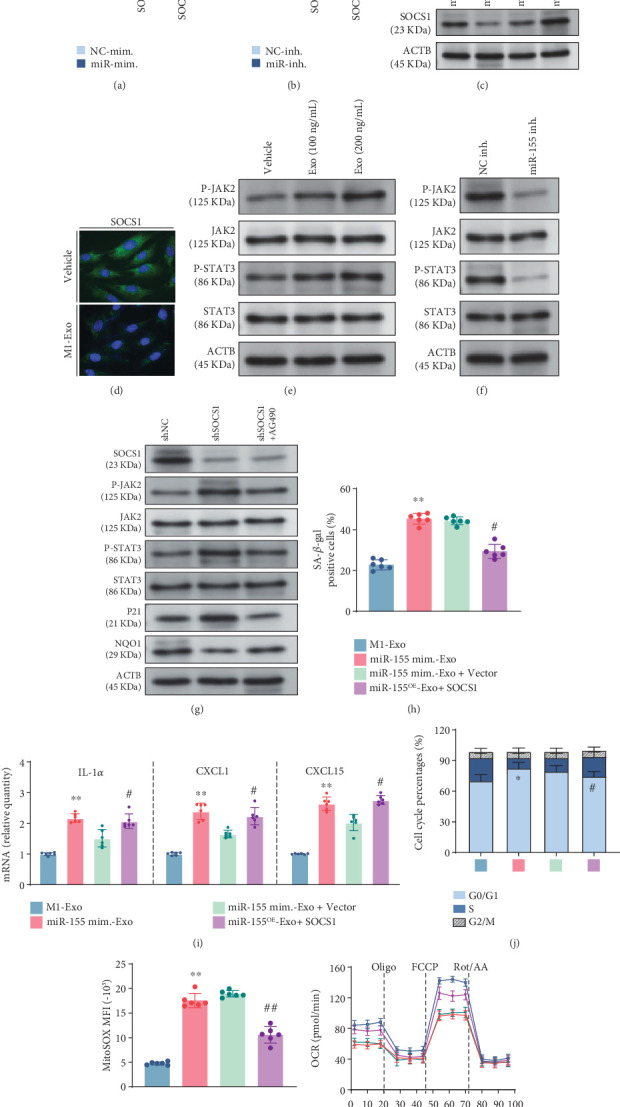
miR-155 loaded in M1 M*φ*-derived exosomes regulates EC senescence by targeting SOCS1/JAK2/STAT3 signaling. (a) Luciferase reporter assays were performed in ECs cotransfected with wild-type or mutant SOCS1 3⁣′UTR and miR-155 mimics (*n* = 6, biological replicates). ⁣^∗^*p* < 0.05. (b) Luciferase reporter assays were performed in ECs cotransfected with wild-type or mutant SOCS1 3⁣′UTR and inhibitors (*n* = 6, biological replicates). ⁣^∗^*p* < 0.05. (c) The protein level of SOCS1 in ECs cocultured with NC mim.-Exo, miR-155 mim.-Exo, NC inh.-Exo, and miR-155 inh.-Exo (exosomes derived from miR-155 mimic or miR-155 inhibitor-treated M1 macrophage). (d) Representative confocal images of SOCS1 staining in ECs cocultured with or without M1-Exo (*n* = 6, biological replicates). (e) Western blot analysis of P-JAK2, JAK2, P-STAT3, and STAT3 in ECs transfected with different concentrations of M1 M*φ*-derived exosomes (100, 200 ng/*μ*L) (*n* = 6, biological replicates). (f) Western blot analysis of P-JAK2, JAK2, P-STAT3, and STAT3 in ECs cocultured with NC mim.-Exo, miR-155 mim.-Exo, NC inh.-Exo, and miR-155 inh.-Exo. (g) Western blot analysis of SOCS1, P-JAK2, JAK2, P-STAT3, STAT3, P21, and NQO1 in ECs cocultured with shRNA-NC, shRNA-SOCS1, and shRNA-SOCS1 with AG490, a selective inhibitor of the JAK2/STAT3 signaling pathway. (h–l) Rescue experiments for miR-155 overexpression were carried out by upregulating SOCS1 in ECs. (h) Quantification of SA*β*G-positive cells. (i) The relative mRNA abundance of key SASP components, IL-1*α*, CXCL1, and CXCL15 (*n* = 6, biological replicates). (j) Cell cycle analysis by flow cytometry was performed (*n* = 6, biological replicates). (k) MitoSOX-indicated mtROS production was determined by flow cytometry (*n* = 6, biological replicates). (l) Mitochondrial O_2_ consumption rate (OCR) was performed using a Seahorse analyzer (*n* = 6, biological replicates). ⁣^∗^*p* < 0.05 and ⁣^∗∗^*p* < 0.01 versus M1-Exo. ^#^*p* < 0.05 and ^##^*p* < 0.01 versus miR-155 mimic-Exo.

To explore the intricate molecular mechanisms by which miR-155 regulates endothelial senescence, we tried to elucidate the candidate target genes regulated by miR-155. Six miRNA-target prediction databases, namely, miRDB, StarBase, TargetScan, miRTarBase, PicTar, and MultiSystem, were employed to predict a set of target genes regulated by miR-155. Results identified three putative target genes of miR-155 (Figure S6A). Notably, treatment with M1-Exo (Figure S6B) or upregulating miR-155 using specific mimics (Figure S6C) resulted in a significant decrease in Socs1 transcripts in ECs. In contrast, the expressions of the other two prospective target genes (Ikbke, Bach1) remained not appreciably altered. Further examination through sequence alignment analysis unveiled a binding site of miR-155 on the 3⁣′UTR of Socs1 transcripts. We constructed luciferase reporter plasmids with wild-type and mutant 3⁣′UTRs of SOCS1 transcripts to validate the potential role of miR-155 in regulating SOCS1 (Figure S6D). The results of dual-luciferase reporter assays clearly demonstrated that the overexpression of miR-155 effectively decreased the luciferase activity of the wild-type SOCS1 3⁣′UTRs, whereas no changes were observed in mutant SOCS1 3⁣′UTRs (Figure 4a). Correspondingly, knockdown of miR-155 elevated the luciferase activity of the wild-type SOCS1 3⁣′UTRs without affecting the mutant SOCS1 3⁣′UTRs (Figure 4b). The results were further substantiated by both western blot analysis (Figure 4c) and immunofluorescent experimentation (Figure 4d), confirming that the overexpression of miR-155 or the administration of M1-Exo engendered a reduction in the protein levels of SOCS1.

Cumulative evidence suggests that exosomal miR-155 exerts biological effects by directly targeting SOCS1 [31, 32]. And SOCS1 binds to JAK2, acting as a specific inhibitor for the JAK2/STAT3 pathway [33–35]. Activation of JAK/STAT has been reported to trigger cell cycle arrest by increasing p21Cip1 expression [36] and mtROS production by decreasing NQO1 expression, a scavenger for ROS [37]. Thus, we speculated that SOCS1 plays a vital role in modulating EC senescence via the JAK2/STAT3-dependent pathway. Western blot analysis revealed that treatment of M1-Exo increased phosphorylation levels of JAK2 and STAT3 in a concentration-dependent manner (Figure 4e with quantification in Figure S7A). Furthermore, stimulation of miR-155 mimic-Exo significantly increased the phosphorylation levels of JAK2 and STAT3 (Figure 4f with quantification in Figure S7B). shRNA-mediated SOCS1 silence resulted in significantly increased phosphorylation levels of JAK2 and STAT3, paralleled with increased p21Cip1 and decreased NQO1 expression. Treatment with AG490, a JAK2/STAT3 inhibitor, alleviated the effect of SOCS1 silence on the phosphorylation of JAK2 and STAT3 (Figure 4g with quantification in Figure S7C). These findings indicate that miR-155 enriched in M1-Exo directly suppresses SOCS1, thereby activating the JAK2/STAT3 signaling.

### 3.6. Exosomal miR-155 Results in Endothelial Senescence Through the SOCS1/JAK2/STAT3 Axis

To confirm whether miR-155 delivered by M1-Exo contributes to EC senescence via modulation of the SOCS1/JAK2/STAT3 pathway, a variety of in vitro loss- and gain-of-function experiments were conducted. We administered miR-155 mimic-Exo into SOCS1-overexpressed ECs and miR-155 inhibitor-Exo into SOCS1-knockdown ECs. Intriguingly, we found that increasing SOCS1 expression partly attenuated the SA*β*G expression (Figure 4h) and the increased expression of SASP components, IL-1*α*, CXCL1, and CXCL15 (Figure 4i) induced by coincubation with miR-155 mimic-Exo. SOCS1 overexpression reversed cell cycle arrest induced by miR-155 mimic-Exo (Figure 4j). Furthermore, upregulation of SOCS1 mitigated the excessive production of mtROS incited by miR-155 mimic-Exo (Figure 4k). Moreover, the aberrant mitochondrial OXPHOS induced by miR-155 mimic-Exo was rescued by overexpression of SOCS1 (Figure 4l and Figure S8). Notably, opposite results were observed in SOCS1-knockdown ECs following administration of miR-155 inhibitor-Exo.

### 3.7. Knockdown of Exosomal miR-155 Rescues Endothelial Dysfunction via Regulating SOCS1

**Figure 5:**
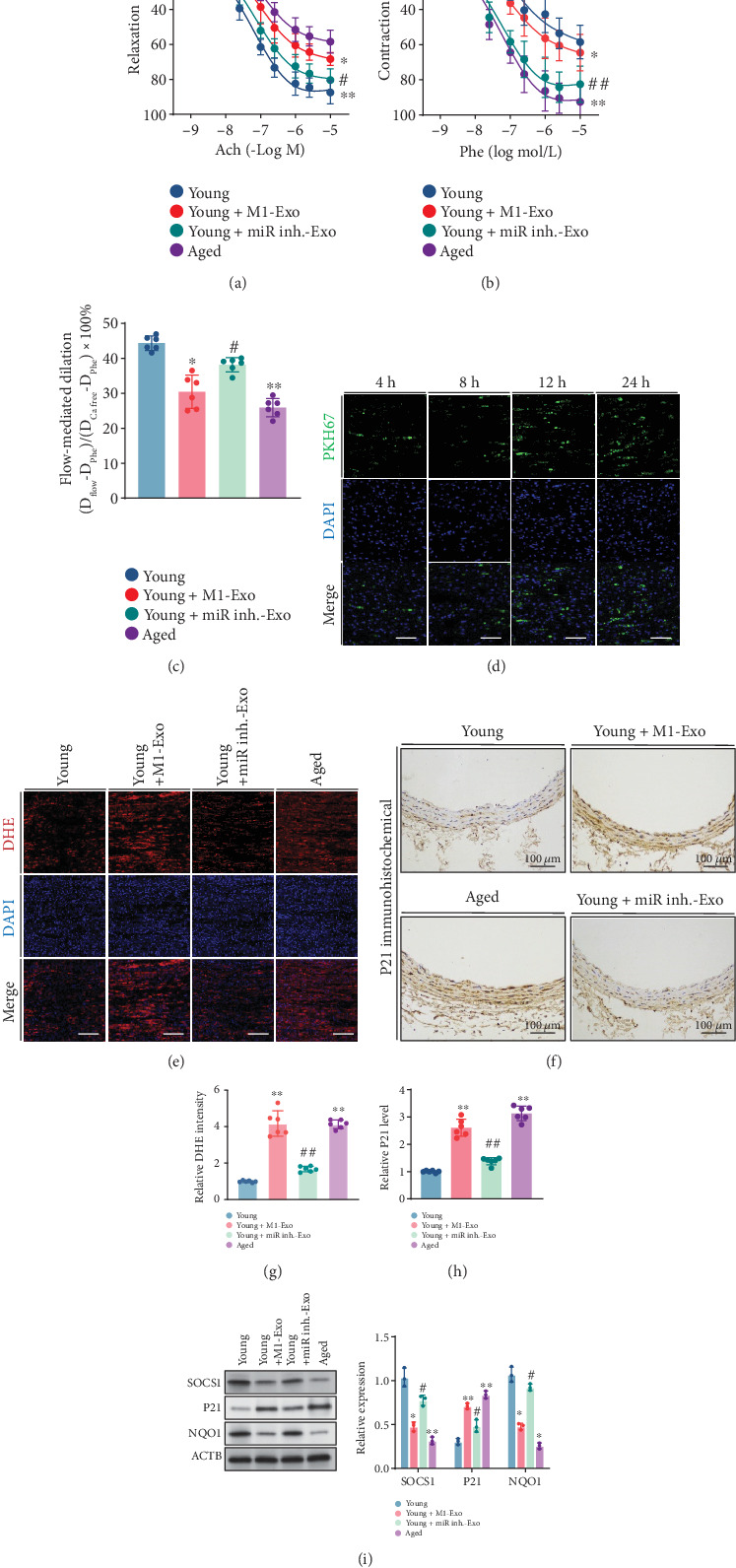
Knockdown of miR-155 rescued M1 M*φ*-derived exosome-induced impairment of endothelial function via regulating SOCS1. Young mice were administrated with PBS, M1-Exo, and miR-155 inhibitor-Exo (exosomes derived from miR-155 inhibitor-treated M1 macrophage) via tail intravenous injection, and old mice were set as control. Endothelial function was measured 2 days after injection. (a) ACh concentration–response curves in the aortas (*n* = 6, biological replicates). (b) Cumulative contraction–response curves to Phe in the aortas (*n* = 6, biological replicates). (c) Flow-mediated dilatation in mesenteric arteries (*n* = 6). (d) *En face* confocal microscopic images showed uptake of exosomes by young mouse aortic endothelial cells in a time-dependent manner. Nuclei stained by DAPI in blue, and exosomes stained by PKH67 in green. Scale bar, 100 *μ*m. (e, g) *En face* immunofluorescence images and quantification of ROS indicated by DHE probe in mouse aortic endothelial cells. The nuclei were stained with DAPI in blue and the DHE signal in red (bar, 20 *μ*m) (*n* = 6, biological replicates). Scale bar, 200 *μ*m. Immunohistological staining of P21 in the aortas (f) and quantification (h). (i) Western blot analysis and quantification of SOCS1, P21, and NQO1 in primary aortic ECs isolated from different groups of mice. ⁣^∗^*p* < 0.05 and ⁣^∗∗^*p* < 0.01 versus young. ^#^*p* < 0.05 and ^##^*p* < 0.01 versus young + M1-Exo.

The prosenescence effects of miR-155 encapsulated within M1-Exo in vitro impelled us to investigate their impact on endothelial function in vivo. We administered M1-Exo and miR-155 inhibitor-Exo to young mice (3-month-old) via tail vein injection. Notably, M1-Exo administration inhibited endothelium-dependent relaxation, whereas knockdown of miR-155 partially restored such adverse effects (Figure 5a). Moreover, aortic rings from young mice treated with M1-Exo displayed significant contraction, which was abolished by miR-155 knockdown (Figure 5b). Similarly, M1-Exo notably impaired FMD in resistance mesenteric arteries, a detrimental effect reversed by miR-155 knockdown (Figure 5c). Immunofluorescence results revealed the incorporation of PKH67-labeled M1-Exo into the ECs of mouse aortas, intensifying with prolonged incubation (Figure 5d). *En face* immunofluorescent staining of DHE, a ROS probe, unveiled increased production of ROS prompted by M1-Exo, which was mitigated by miR-155 downregulation (Figure 5e,g). Immunohistochemical analysis highlighted a significant rise in P21 expression in aortic tissues post M1-Exo administration. By contrast, miR-155 inhibitor-Exo had no discernible effect on P21 expression (Figure 5f,h). Furthermore, primary aortic ECs were isolated from different experimental groups. Western blotting analysis showed decreased expression of SOCS1 and NQO1, along with elevated P21 levels, following M1-Exo administration (Figure 5i). These results underscored the role of miR-155 knockdown in mitigating the deleterious impact of M1-Exo on endothelial function through its regulation of SOCS1.

## 4. Discussion

Our study showed that the Exo derived from M1-polarized M*φ*, which infiltrated the aortas of aged mice, aggravated endothelial senescence via inducing cell cycle arrest and mitochondrial oxidative damage. The prosenescence effect is attributable to the enclosed miR-155 in the Exo, targeting SOCS1 in ECs and activating the JAK2/STAT3 signaling.

Advanced aging is a major global health challenge and represents a critical driver of CVD pathogenesis [2]. Endothelial dysfunction, a prominent age-related arterial condition, likely contributes to CVD development. Recent studies highlight chronic, sterile, low-grade inflammation as a hallmark of aging, which plays a pivotal role in various vascular pathologies, ranging from endothelial dysfunction and atherogenesis to aneurysm formation [4, 7]. Immune cell infiltration, especially M*φ*, is proposed as a primary source of this inflammatory microenvironment within the vessel wall during aging [38–40]. The proinflammatory microenvironment triggers endothelial dysfunction and substantially contributes to the pathogenesis of vascular diseases. Our study demonstrated a conspicuous accumulation of M1 M*φ* within the arterial wall of aged mice, concomitant with impaired endothelial function, indicating a compelling correlation between M1 M*φ* infiltration and age-related endothelial dysfunction. Age-related changes in M*φ* function likely involve shifts along multiple axes of activation rather than simple M1/M2 switching, reflecting the complex remodeling of immune responses in age-related vasculature [11], which warranted further exploration in future studies.

Senescent ECs in aging vasculature are crucial for vascular endothelial dysfunction, leading to the onset and progression of CVD [2, 41]. EC senescence alters vascular structure and function. However, the predisposing factors and regulatory mechanisms governing EC senescence remain incompletely understood. Inflammation is a critical initiator of age-related vascular pathology, and it is reasonable to surmise that M1 M*φ* infiltration in the arterial wall might contribute to endothelial senescence. Activated M*φ* modulate the immune response through direct intercellular contact and the secretion of the secretome, such as cytokines and Exo. The multifaceted functions of M*φ*-Exo in various diseases have been widely investigated [18, 19, 42, 43]. We conducted a series of experiments to validate the capacity of M1-Exo to induce EC senescence. This was supported by increased SA*β*G levels, elevated production of SASP key components, and upregulation of senescent makers. Notably, administration of M1-Exo to young mice elicited endothelial dysfunction in the aortas, similar to the manifestations observed in aged mice.

The most representative characteristic of senescent cells is their irreversible cell cycle arrest, evidenced by an increased presence in the G0/G1 phases, leading to impaired cellular function and regeneration. P21Cip1 functions as an inhibitor of cyclin-dependent kinases and plays a critical role in the regulation of senescence-mediated cell cycle arrest [23]. Another hallmark of aging is the heightened generation of ROS, causing mitochondrial damage and contributing to age-related endothelial dysfunction. Mitochondrial oxidative damage, particularly the deterioration of OXPHOS, has been extensively implicated in cellular senescence [22]. In the present study, upregulation of p21Cip1 accompanied by pronounced cell cycle arrest, excessive mtROS generation, and abnormalities in OXPHOS was observed in ECs following coculture with M1-Exo. Our results showed that M1-Exo instigated endothelial senescence by triggering cell cycle arrest and mitochondrial damage.

Exo-mediated miRNAs shuttling is essential for intercellular communication [16, 17]. Exo from M1 M*φ* are widely known to contribute to inflammatory processes and tissue injury through transporting miRNAs. Through bioinformatics analysis and experimental validation, we confirmed the upregulation of miR-155 in M1 M*φ* and M1-Exo. We further found that the prosenescence impact exerted by M1-Exo predominantly hinged upon the transmission miR-155 into ECs. Intriguingly, the harmful consequences of M1-Exo administration to young mice, inducing endothelial dysfunction, were effectively abolished by the miR-155 knockdown within M1-Exo. Accumulated evidence identified the critical role of miR-155 in regulating the effects of Exo on target cells. Exosomal miR-155 derived from M*φ* has been demonstrated to transfer into cardiac fibroblasts, leading to cardiac inflammation. Attenuating miR-155 expression ameliorated the proinflammatory impact of M*φ* in ulcerative colitis. Ge et al. proposed that exosomal miR-155 derived from M1 M*φ* facilitates EndoMT and disrupts mitochondrial function in traumatic spinal cord injury [44]. Our results demonstrated that miR-155 is involved in M1-Exo-mediated endothelial senescence via regulating cell cycle and mitochondrial oxidative homeostasis. Knockdown of miR-155 eliminated the detrimental effect of M1-Exo on endothelial function in vivo.

To investigate the intricate mechanisms underlying the role of exosomal miR-155, we aimed to identify downstream target genes regulated by miR-155. We unequivocally identified socs1 as a direct target gene of miR-155 using online databases, sequence alignment studies, and dual-luciferase reporter assays. Several independent studies indicated that exosomal miR-155 exerts biological effects by directly targeting SOCS1 [31, 32]. Among the members of the SOCS family, SOCS1 is exceptionally potent, selectively impeding JAK2 kinase activity and attenuating phosphorylation of STAT3 [33–35]. Zhou et al. elucidated that exosomal miR-155 derived from melanoma cells facilitates a proangiogenic transition in cancer-associated fibroblasts through an activation of SOCS1/JAK2/STAT3 cascades [32]. Furthermore, miR-155 derived from colorectal cancer Exo stimulates the activation of fibroblasts through the SOCS1/JAK2/STAT3 signaling pathway [42]. Notably, JAK2/STAT3 signal regulates cellular senescence via p21. Hawthorne et al. confirmed STAT3 binds to the p21Cip1 promoter [45]. Xu et al. reported inhibiting the JAK/STAT pathway mitigates SASP in preadipocytes and ECs [46]. Meanwhile, STAT signaling potentiates oxidative stress via downregulating NQO1, a key ROS scavenger [46, 47]. Our results unequivocally demonstrated that miR-155 transfer via M1-Exo profoundly dampened SOCS1 expression in ECs with subsequent JAK2/STAT3 signaling activation. Activating JAK2/STAT3 signaling mediated by knockdown of SOCS1 in ECs resulted in increased p21Cip1 and a concomitant decrease in NQO1 expression.

In summary, the current study demonstrated for the first time that infiltrating M1-polarized M*φ* induce endothelial senescence by triggering cell cycle arrest and mitochondrial oxidative stress, expedited by the transmission of Exo harboring miR-155. Exo-dependent transfer of miR-155 into ECs inhibited SOCS1 expression and activated JAK2/STAT3 signaling, resulting in the augmentation of P21 and the concomitant attenuation of NQO1. Our findings provide unique insights into the interplay between the proinflammatory milieu in the aging arterial wall and the onset of endothelial senescence, highlighting a potential mechanism that underlies the pathological advancement of age-related vascular diseases.

## Data Availability

The data that support the findings of this study are available on request from the corresponding authors. The data are not publicly available due to privacy or ethical restrictions.
