# Supplementary material for: M1 Macrophage is a Novel Potential Trigger for Endothelial Senescence: Role of Exosomal miR-155 Targeting SOCS1 Signal
Source: Hum Mutat. 2025 May 30;2025:6771390. doi: 10.1155/humu/6771390 (PMC12143949; doi:10.1155/humu/6771390)
Supplement: Supporting Information — Additional supporting information can be found online in the Supporting Information section. [file 6771390.f1.zip › Supplementary figures.pdf]

Figure S1

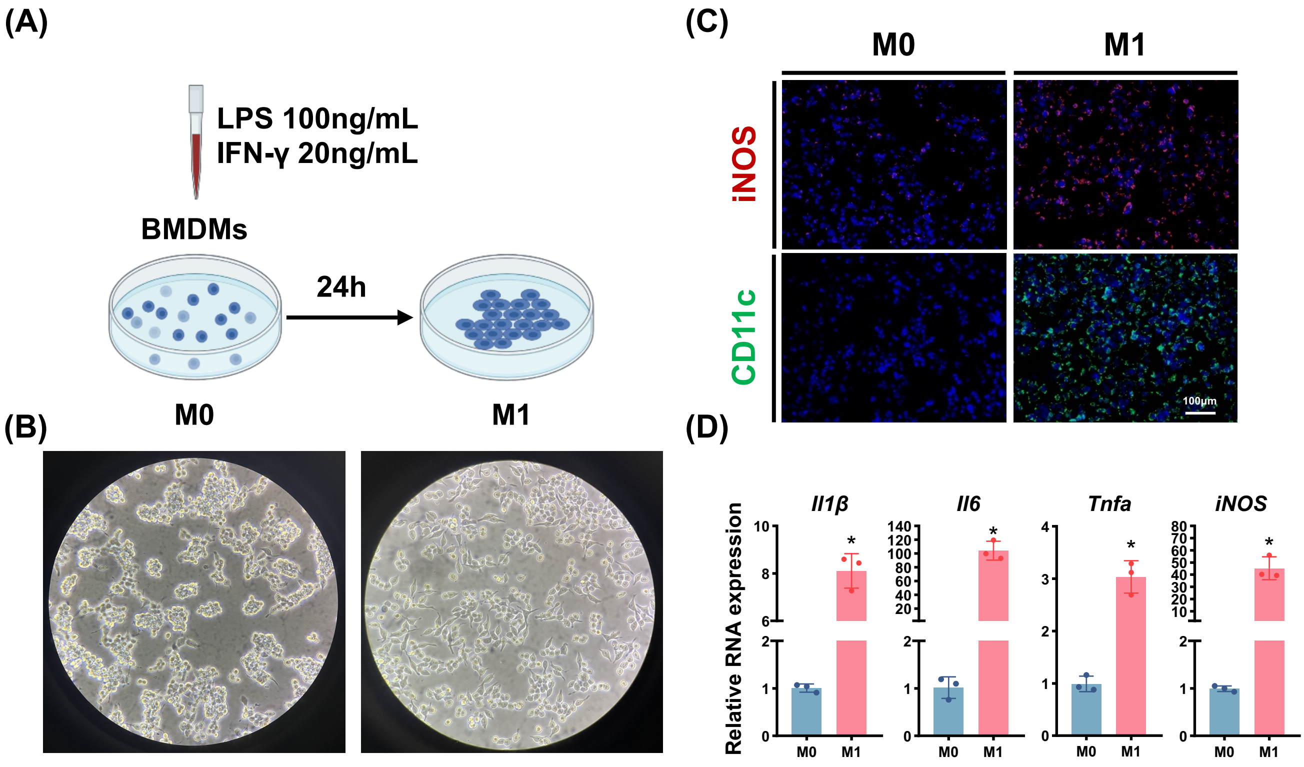

Figure S1.
(A) M1 polarized macrophages were induced from BMDMs by 24h-stimulation of LPS and IFN-γ. (B) Morphology of BMDMs (M0) and M1 macrophages under light microscope.(C) Representative immunofluorescent images of iNOS and MHC-II in M0 and M1 macrophages. Scale bar, 100μm. (D) Relative RNA level of IL-1β, IL-6, TNF-α and iNOS in M0 and M1 macrophages. *p < 0.05.

Figure S2

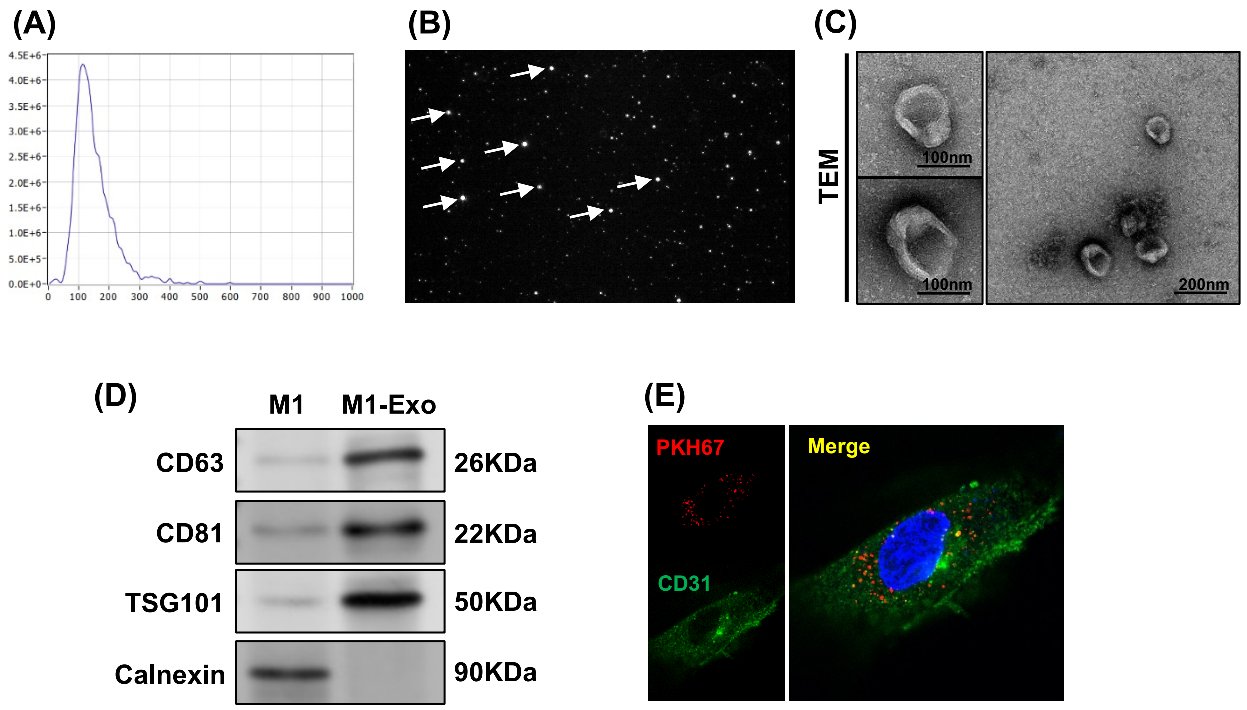

Figure S2.
(A and B) M1 macrophage-derived exosomes were investigated by Nanoparticle Tracking Analyses (NTA); the white arrows indicate small particles moving in Brownian motion. (C) TEM images of the isolated exosomes from M1 Macrophage (M1-Exo) (n = 3). (D) Western blot analysis of Exosome fractions and cell lysates of M1 Macrophage with antibodies against exosomal proteins (CD63, CD81, TSG101, Calnexin) and the cellular protein calnexin (n = 3). (E) Confocal image showed the uptake of M1-Exo by ECs. The exosomes were tagged with PKH67 (n = 3).

Figure S3

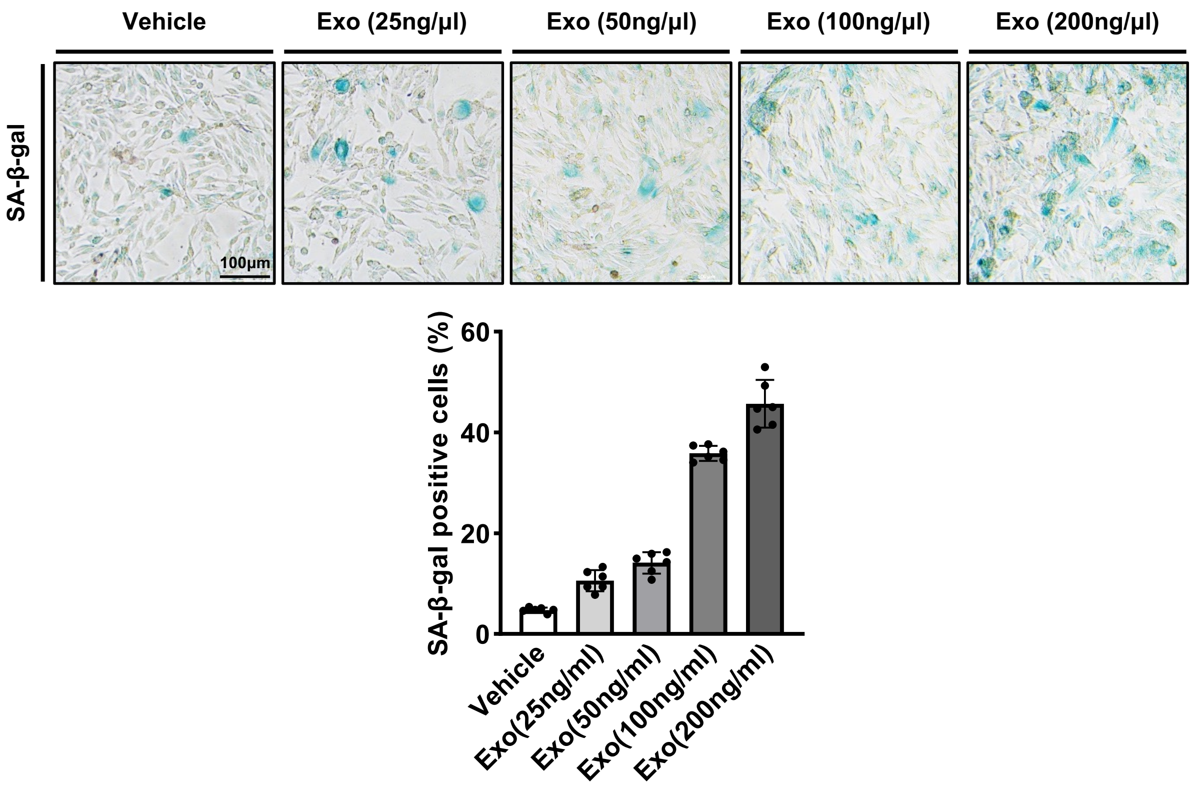

Figure S3.

SA-β-gal level in endothelial cells co-cultured with different concentrations of M1 macrophages-derived exosomes. Scale bar, 100μm. SA-β-gal, Senescence-associated-β-galactosidase. Exo, exosome.

Figure S4

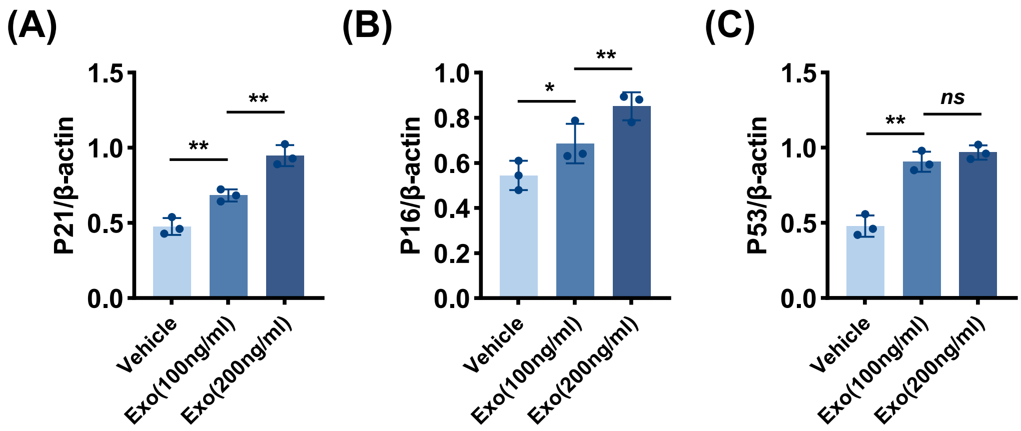

Figure S4.
Quantification of western blotting images of the senescence markers P16 (A), P21 (B), P53 (C) in ECs with different concentrations of M1-Exo (100 ng/μl, 200 ng/μl) co-incubation (n = 3). *p < 0.05. **p < 0.01. ns, no significant.

Figure S5

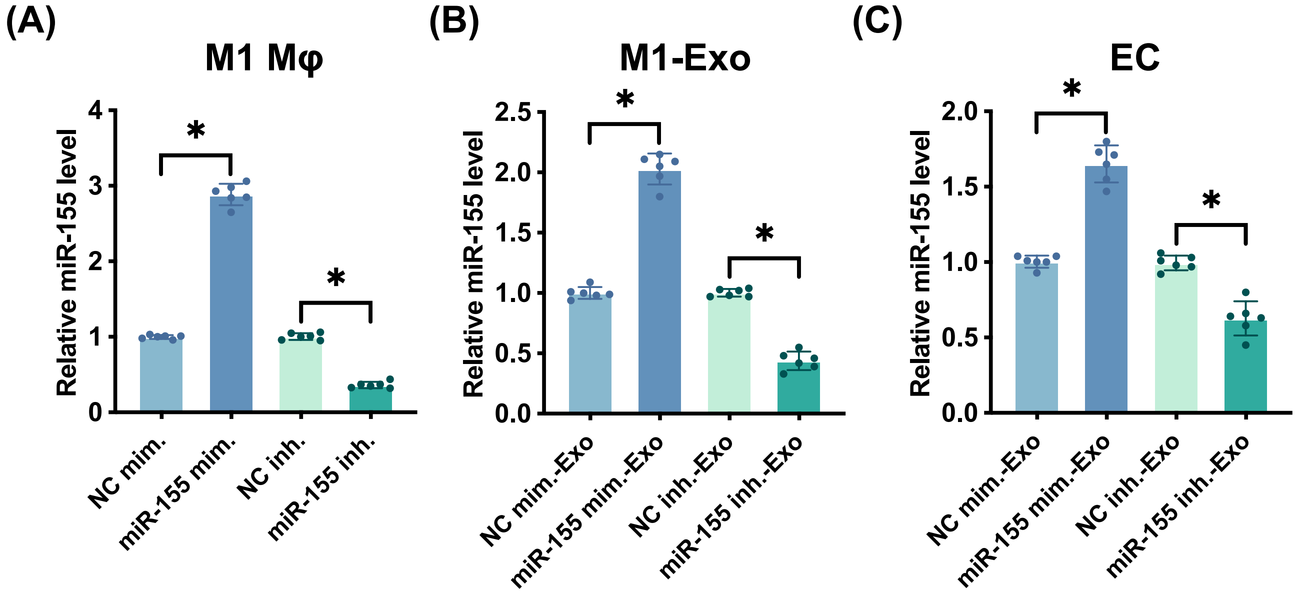

Figure S5.
(A) Transfection efficiency of miR-155 overexpression and knockdown in M1 macrophages. (B) The relative expression of miR-155 in exosomes derived from M1 macrophages in indicated groups. (C) The relative expression of miR-155 in endothelial cells administered with NC mim.-Exo, miR-155 mim.-Exo, NC inh.-Exo, and miR-155 inh.-Exo. Exo, exosome. $^*p < 0.05$.

Figure S6

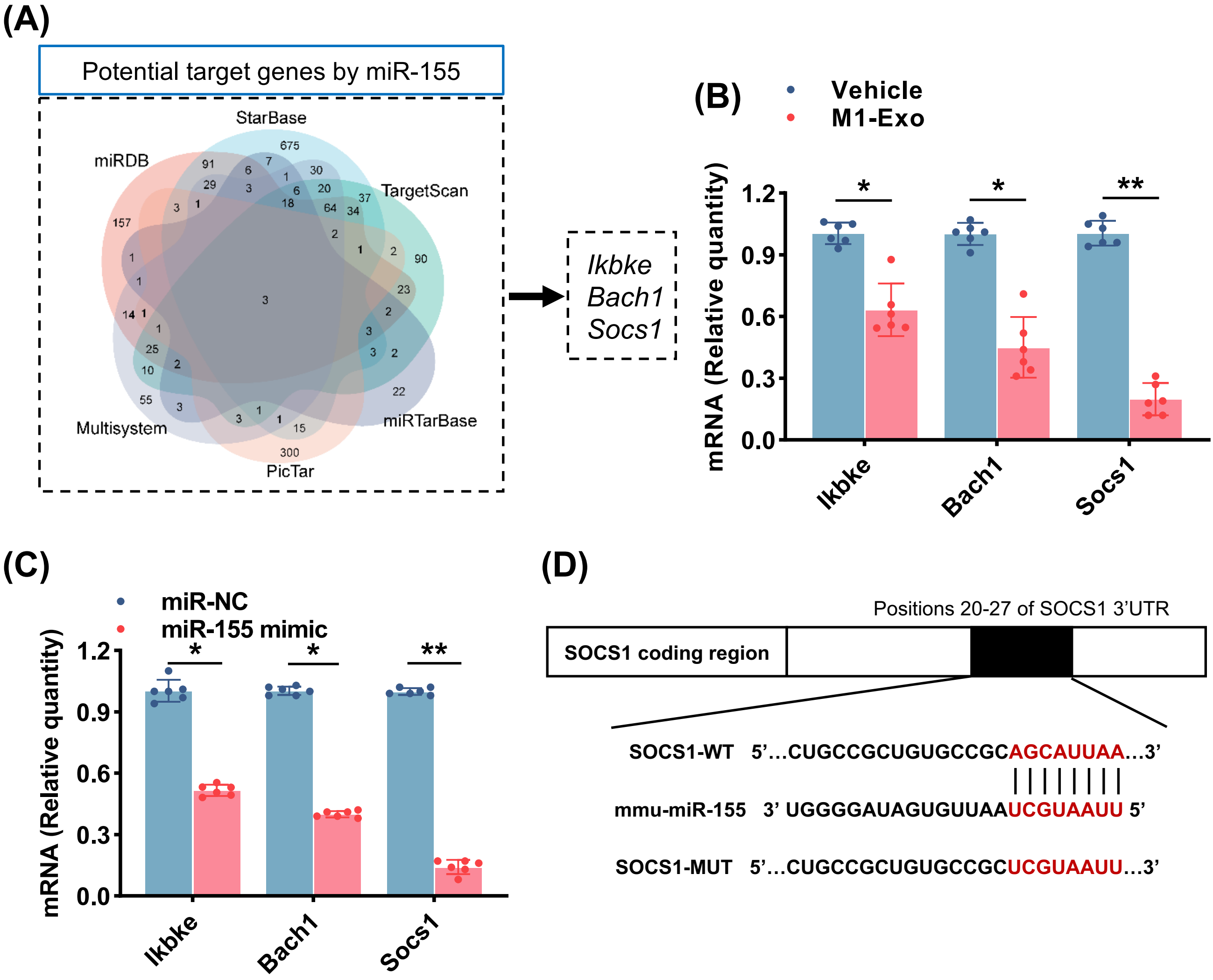

Figure S6.
(A) Four miRNA-target gene prediction databases including StarBase, TargetScan, miRTarBase, PicTar, Multisystem, and miRDB were used to identify potential miR-155 targets. (B) The mRNA expression of three potential predicted targets of miR-155 was analyzed by qRT-PCR in endothelial cells treated with or without M1 macrophage-derived Exo (n = 6). *p < 0.05. (C) The mRNA expression of three potential predicted targets of miR-155 was analyzed by qRT-PCR in endothelial cells treated with 20 nM miR-155 mimics or NC mimics (n = 6). *p < 0.05. (D) miR-155 regulated SOCS6 by directly targeting the 3'-UTR. Exo, exosome.

Figure S7

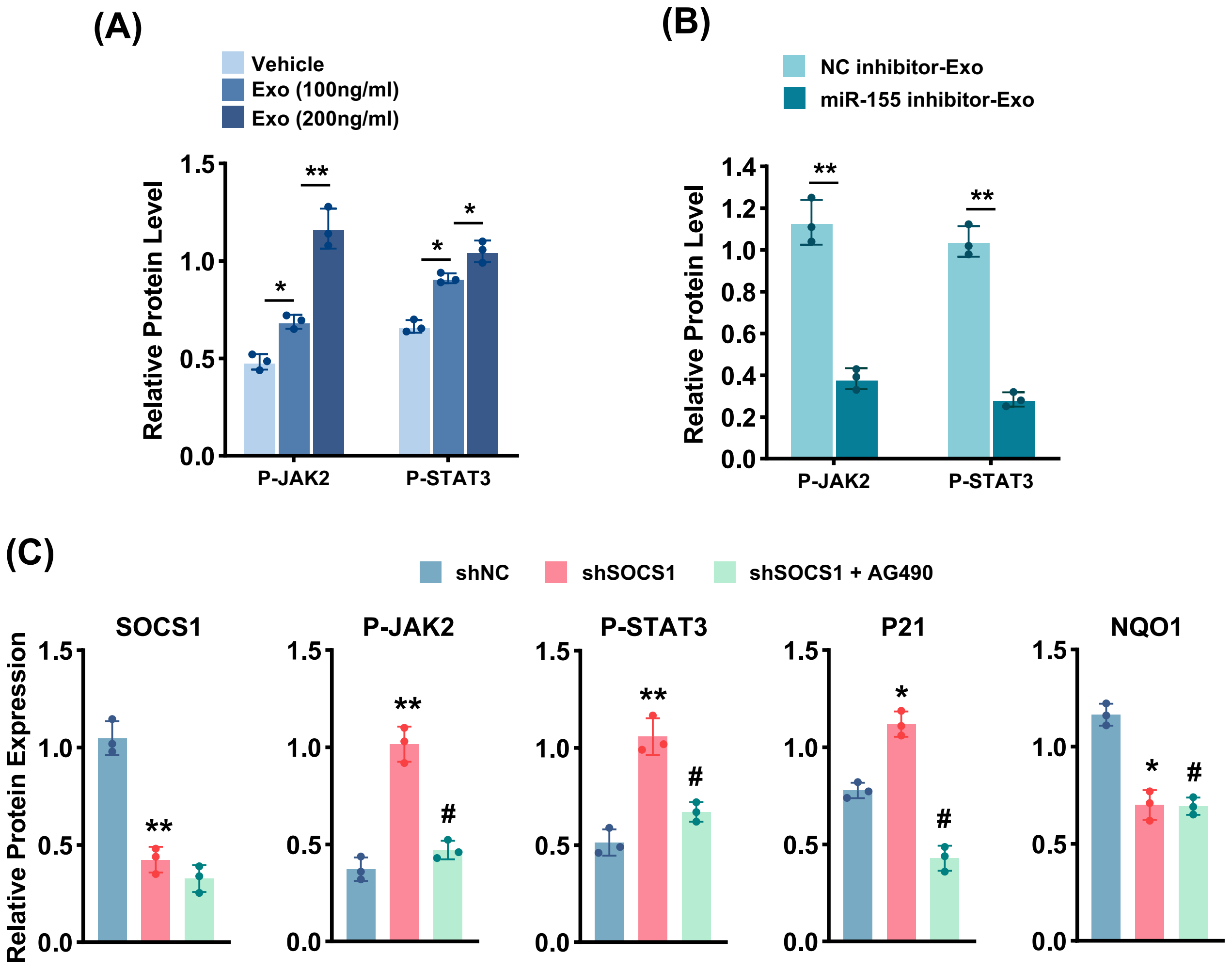

Figure S7.
(A) Quantification of protein level of P-JAK2/JAK2 and P-STAT3/STAT3 in ECs with different concentrations of M1 Mφ-derived Exo (100 ng/μl, 200 ng/μl) co-incubation (n = 3). $*p < 0.05$. $**p < 0.01$. (B) Quantification of protein level of P-JAK2/JAK2 and P-STAT3/STAT3 in ECs co-cultured with NC inhibitor-Exo (Exosomes from M1 Mφ treated with NC inhibitor) and miR-155 inhibitor-Exo (Exosomes from M1 Mφ treated with miR-155 inhibitor) (n = 3). $**p < 0.01$. (C) Quantification of protein level of SOCS1, P-JAK2/JAK2, P-STAT3/STAT3, P21, and NQO1 in ECs co-cultured with nontargeting shRNA (shNC), SOCS1 shRNA (shSOCS1), or SOCS1 shRNA with JAK2 inhibitor AG490 (n = 3). Exo, exosome. $*p < 0.05$ vs shNC. $**p < 0.01$ vs shNC. $\#p < 0.05$ vs shSOCS1.

Figure S8

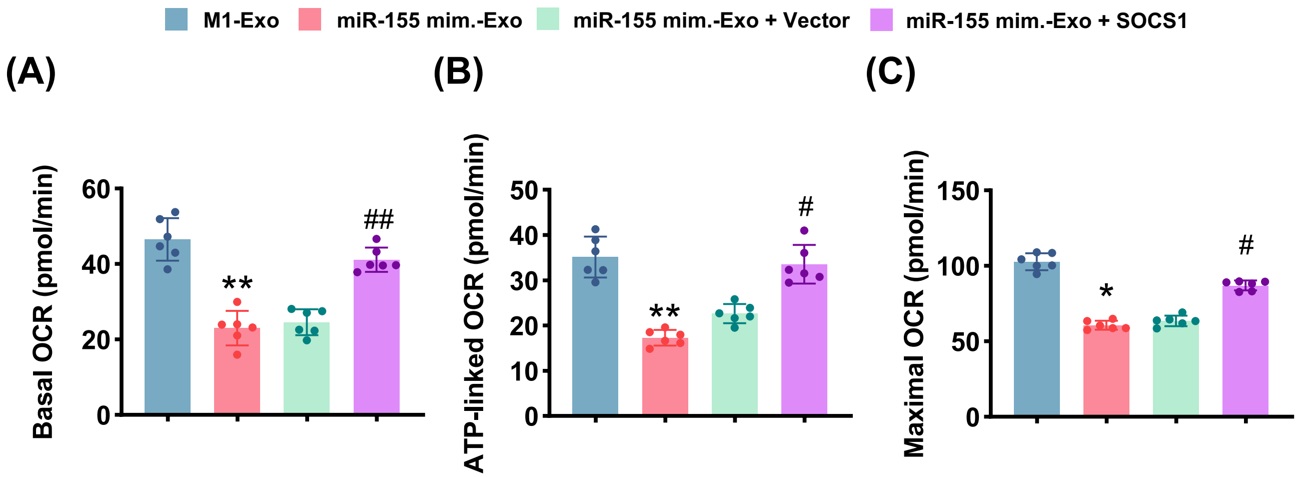

Figure S8.

Rescue experiments for miR-155 overexpression were carried out by upregulating SOCS1 in endothelial cells. Quantification of basal (A), ATP-linked (B) and maximal OCR (C) detected by using a Seahorse analyzer (n = 6). Exo, exosome. *p < 0.05 vs M1-Exo. **p < 0.01 vs M1-Exo. #p < 0.05 vs miR-155 mim.-Exo + Vector. ##p < 0.01 vs miR-155 mim.-Exo + Vector.
