# Supplementary material for: M1 Macrophage is a Novel Potential Trigger for Endothelial Senescence: Role of Exosomal miR-155 Targeting SOCS1 Signal
Source: Hum Mutat. 2025 May 30;2025:6771390. doi: 10.1155/humu/6771390 (PMC12143949; doi:10.1155/humu/6771390)
Supplement: Supporting Information — Additional supporting information can be found online in the Supporting Information section. [file 6771390.f1.zip › Supplementary Tables.docx]

| **Primer Name** | **Primer** | **Sequence** |
| --- | --- | --- |
| Mature mmu-miR-155-5p | Forward | AATGCTAATTGTGATAGGGG |
|  | Reverse | GAACATGTCTGCGTATCTC |
| CD68 | Forward | GGCGGTGGAATACAATGTGTCC |
|  | Reverse | AGCAGGTCAAGGTGAACAGCTG |
| CD80 | Forward | CCTCAAGTTTCCATGTCCAAGGC |
|  | Reverse | GAGGAGAGTTGTAACGGCAAGG |
| CD86 | Forward | ACGTATTGGAAGGAGATTACAGCT |
|  | Reverse | TCTGTCAGCGTTACTATCCCGC |
| iNOS | Forward | GAGACAGGGAAGTCTGAAGCAC |
|  | Reverse | CCAGCAGTAGTTGCTCCTCTTC |
| MHC-II | Forward | GCTGTGGACAAAGCTAACCTGG |
|  | Reverse | AGGTTCACAGGGCTTCTGGAGA |
| IL-1a | Forward | ACGGCTGAGTTTCAGTGAGACC |
|  | Reverse | CACTCTGGTAGGTGTAAGGTGC |
| IL-1β | Forward | TGGACCTTCCAGGATGAGGACA |
|  | Reverse | GTTCATCTCGGAGCCTGTAGTG |
| IL-6 | Forward | TACCACTTCACAAGTCGGAGGC |
|  | Reverse | CTGCAAGTGCATCATCGTTGTTC |
| CXCL15 | Forward | GGTGATATTCGAGACCATTTACTG |
|  | Reverse | GCCAACAGTAGCCTTCACCCAT |
| TNF-a | Forward | GGTGCCTATGTCTCAGCCTCTT |
|  | Reverse | GCCATAGAACTGATGAGAGGGAG |
| MCP-1 | Forward | GCTACAAGAGGATCACCAGCAG |
|  | Reverse | GTCTGGACCCATTCCTTCTTGG |
| CXCL-1 | Forward | TCCAGAGCTTGAAGGTGTTGCC |
|  | Reverse | AACCAAGGGAGCTTCAGGGTCA |
| Ikbke | Forward | CCCAAAGTTCGTCCCTAAGGTTG |
|  | Reverse | ATCAACGCCTGTCCATCCAGCA |
| Bachl | Forward | CCATGACATCCGCAGAAGGAGT |
|  | Reverse | GCGTTGACAGAATGTGGTCTCG |
| SOCS1 | Forward | AGTCGCCAACGGAACTGCTTCT |
|  | Reverse | GTAGTGCTCCAGCAGCTCGAAA |

Table S1. Primer sequences used for qPCR

Table S2. Primary antibodies used for western blot, flow cytometry, or immunostaining.

| **Primary antibodies** | | | | |
| --- | --- | --- | --- | --- |
| **Target protein** | **Species** | **Supplier** | **Catalogue number** | **RRID** |
| CD68 | Rabbit | CST | #26042 | AB_2920587 |
| CD45-FITC | Rat | BD Pharmingen | 553079 | AB_394609 |
| F4/80-BV421 | Rat | BD Pharmingen | 565411 | AB_2734779 |
| CD11c-APC | Hamster | BD Pharmingen | 550261 | AB_398460 |
| CD206-PE | Rat | BD Pharmingen | 568273 | AB_2916867 |
| iNOS | Rabbit | Abcam | ab178945 | AB_2861417 |
| CD11c | Rabbit | CST | #39143 | AB_2924836 |
| CD63 | Rabbit | Abcam | ab217345 | AB_2754982 |
| CD81 | Rabbit | Abcam | ab109201 | AB_10866464 |
| TSG101 | Rabbit | CST | #72312 | AB_2927716 |
| Calnexin | Rabbit | CST | #2679 | AB_2228381 |
| P21Cip1 | Rabbit | CST | #37543 | - |
| P16 INK4A | Rabbit | CST | #29271 | - |
| P53 | Rabbit | CST | #32532 | AB_2757821 |
| SOCS1 | Rabbit | Abcam | ab280895 | - |
| P-JAK2 | Rabbit | CST | #3771 | AB_330403 |
| JAK2 | Rabbit | CST | #3230 | AB_2128522 |
| P-STAT3 | Rabbit | CST | #9145 | AB_2491009 |
| STAT3 | Rabbit | CST | #12640 | AB_2629499 |
| NQO1 | Rabbit | CST | #62262 | AB_2799623 |
| β-actin (ACTB) | Rabbit | CST | #4970 | AB_2223172 |
